# Epitope-loaded nanoemulsion delivery system with ability of extending antigen release elicits potent Th1 response for intranasal vaccine against *Helicobacter pylori*

**DOI:** 10.1186/s12951-019-0441-y

**Published:** 2019-01-19

**Authors:** Yun Yang, Li Chen, Hong-wu Sun, Hong Guo, Zhen Song, Ying You, Liu-yang Yang, Ya-nan Tong, Ji-ning Gao, Hao Zeng, Wu-chen Yang, Quan-ming Zou

**Affiliations:** 1 National Engineering Research Center of Immunological Products, Department of Microbiology and Biochemical Pharmacy, College of Pharmacy, Third Military Medical University, Chongqing, China; 2 Department of Blood Transfusion, The Second Affiliated Hospital, Third Military Medical University, Chongqing, China; 3 Department of Gastroenterology, The Second Affiliated Hospital, Third Military Medical University, Chongqing, China; 4 Institute of Combined Injury of PLA, College of Military Preventive Medicine, Third Military Medical University of Chinese PLA, Chongqing, China; 5 Department of Hematology, The Second Affiliated Hospital, Third Military Medical University, Chongqing, China

**Keywords:** Nanoemulsion, Epitope vaccine, *Helicobacter pylori*, Intranasal, HpaA

## Abstract

**Background:**

*Helicobacter pylori* (*H. pylori*) infection remains a global public health issue, especially in Asia. Due to the emergence of antibiotic-resistant strains and the complexity of *H. pylori* infection, conventional vaccination is the best way to control the disease. Our previous study found that the *N*-acetyl-neuroaminyllactose-binding hemagglutinin protein (HpaA) is an effective protective antigen for vaccination against *H. pylori* infection, and intranasal immunization with the immunodominant HpaA epitope peptide (HpaA 154-171, P22, MEGVLIPAGFIKVTILEP) in conjunction with a CpG adjuvant decreased bacterial colonization in *H. pylori*-infected mice. However, to confer more robust and effective protection against *H. pylori* infection, an optimized delivery system is needed to enhance the P22-specific memory T cell response.

**Results:**

In this study, an intranasal nanoemulsion (NE) delivery system offering high vaccine efficacy without obvious cytotoxicity was designed and produced. We found that this highly stable system significantly prolonged the nasal residence time and enhanced the cellular uptake of the epitope peptide, which powerfully boosted the specific Th1 responses of the NE-P22 vaccine, thus reducing bacterial colonization without CpG. Furthermore, the protection efficacy was further enhanced by combining the NE-P22 vaccine with CpG.

**Conclusion:**

This epitope-loaded nanoemulsion delivery system was shown to extend antigen release and elicit potent Th1 response, it is an applicable delivery system for intranasal vaccine against *H. pylori*.
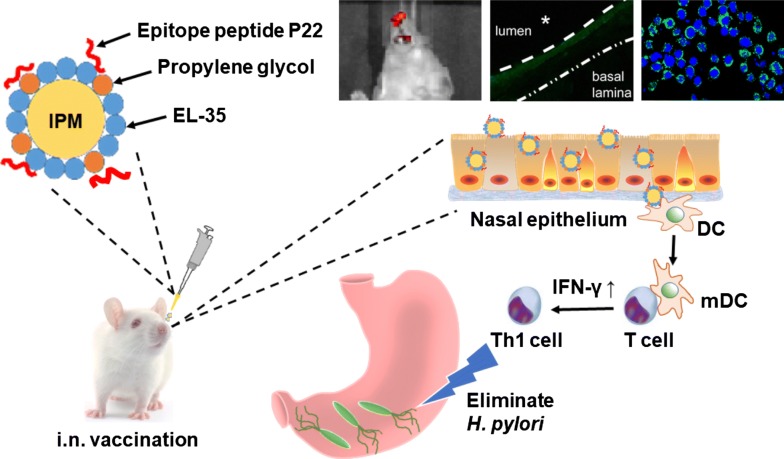

**Electronic supplementary material:**

The online version of this article (10.1186/s12951-019-0441-y) contains supplementary material, which is available to authorized users.

## Introduction

*Helicobacter pylori* (*H. pylori*) causes a common chronic infection that currently affects at least 50% of the global population [1]. *H. pylori* not only causes gastric diseases such as peptic ulcers, chronic gastritis, and gastric adenocarcinoma [2], but also causes cardiovascular [3], pulmonary [4], and haematological diseases [5]. Considering the development of antibiotic resistance and the growing complexity of *H. pylori* therapy, the development of an *H. pylori* vaccine is needed [6].

Recently, we found that HpaA is an effective protective antigen for vaccination against *H. pylori* infection, and intranasal immunization with the immunodominant epitope peptide of HpaA (HpaA 154-171, P22, MEGVLIPAGFIKVTILEP) in conjunction with CpG reduced *H. pylori* colonization in infected mice [7]. HpaA is a surface lipoprotein [8, 9] that is one of the most important colonization factors for *H. pylori* [10] because it is highly conserved [11] and specific [12] to *H. pylori*. Given these characteristics, HpaA has been proposed as a promising candidate antigen for *H. pylori* vaccines [13, 14]. However, to confer more robust protection against *H. pylori* infection, the effectiveness of the vaccine must be improved. As synthetic epitope peptides are not highly immunogenic by themselves due to their low molecular weight [15], a proper delivery system must be developed to enhance the Th1 responses stimulated by P22 and to confer more robust protection against *H. pylori* infection.

Nasal administration is a safe and effective method for vaccine delivery due to its convenience and avoidance of the parenteral route, which increases patient compliance [16]. A recent study described a nanotechnology method to generate nanoemulsions of particles with an average diameter of 1–100 nm using a surfactant or co-surfactant, oil and water. Such nanoemulsions are highly thermodynamically stable, optically isotropic liquids, and they may exhibit robust and extensive anti-microbial, antiviral and antifungal activities [17]. Moreover, these nanoemulsions (NEs) may provide effective adjuvant activity for vaccine products, especially those consisting of intact viruses such as respiratory syncytial influenza or HIV [18, 19]. Our recent study indicated that a NE-based adjuvant potently and powerfully induced broad mucosal immune responses after nasal administration [20]. Thus, NE system with these outstanding properties could be used as the *H. pylori* epitope vaccine carrier for nasal administration.

In this study, we designed and prepared an optimized intranasal epitope-loaded NE delivery system for *H. pylori* vaccine based on screening of the different Smix (Surfactant: Co-surfactant, w/w) ratios and peptide concentrations. Subsequently, the basic characteristics, stability, and cytotoxicity of this NE system were tested. We next tried to systematically examine the ability of the NE system to immune effect. The cellular uptake, induction of dendritic cell maturation, release profile and mucosal retention of this NE delivery system were investigated in vivo and in vitro. The vaccine-induced specific Th1 responses, bacterial colonization, pathological inflammation of gastric tissue were determined in vivo using *H. pylori* infection mice model.

## Materials and methods

### Animals

Six- to eight-week-old SPF female BALB/c mice were purchased from the Experimental Animal Center of the Third Military Medical University. Animal maintenance and experimental procedures were carried out in accordance with the National Institutes of Health Guidelines for the Use of Experimental Animals. All in vivo experiments were approved by the Medicine Animal Care Committee of the Third Military Medical University.

### Preparation of the intranasal NE delivery system

#### Influence of the surfactant-to-oil ratio on the size and zeta potential

To identify an optimal NE formula suitable for this novel system, the influence of the surfactant-to-oil ratio was assessed based on the results of our previous study [21]. Briefly, we chose isopropyl myristate (IPM) as the oil phase, EL-35^®^ (BASF, Germany) as the surfactant and propylene glycol as the co-surfactant, with water as the aqueous phase. The surfactant and the co-surfactant were mixed (Smix) in different mass ratios (2:1, 3:1, 4:1, 5:1, and 6:1) and were then added dropwise to the aqueous phase with gentle agitation. After being equilibrated (i.e., when they showed low viscosity and a clear appearance), the average size, zeta potential and polydispersity index (PDI) of each NE were measured with a Nano ZS (Malvern Instruments Corp., UK) at 25 °C.

#### Influence of the P22 concentration on the size, zeta potential and encapsulation efficacy

To determine the optimum epitope peptide loading capacity, six different concentrations (0, 250, 500, 1000, 1500, and 2000 μg/mL) were examined. NEs for each of the six concentrations were prepared using our previously described method (Smix = 4:1). The average size and zeta potential were then measured as described above. The encapsulation efficacy of these six concentrations was determined by HPLC (E2695, Waters, MA, USA) with a ZORBAX SB-C18 Chromolith column (5 μm, 4.6 mm × 250 mm) at a wavelength of 220 nm according to our previously described method [21].

### Preparation of the NE delivery system

According to the optimal Smix and epitope concentrations, the optimized NE system was prepared. Briefly, Smix ratio was 4:1 (w/w). Next, IPM was added to the Smix to obtain the desired ratio of 9:1 (Smix-IPM). Subsequently, this mixture was added dropwise under gentle agitation to the aqueous phase, which contained the epitope peptide (P22, 1000 μg/ml) and CpG (1000 μg/ml, Invitrogen, Shanghai, China). To prepare a blank NE or a vaccine without CpG, the same protocol was adopted except the peptide or CpG were replaced by distilled water as the aqueous phase.

### Basic characteristics and stability of the novel NE system

The morphology of the NE particles was observed by transmission electron microscopy (TEM) as previously described [20]. Briefly, 10 μL of sample (diluted 100-fold with distilled water) was dropped on a 100-mesh carbon copper grid. Samples were observed with an FEI TECNAI10 TEM (Philips Electron Optics, Holland) at a voltage of 120 kV. The molecular morphology of the NE particles was observed on an IPC-208B atomic force microscope (AFM; Chongqing University, China) over a scanned area of 420 × 420 nm using a point-by-point scanning method as previously described [20]. The stability of the P22 was determined by matrix-assisted laser desorption/ionization time-of-flight mass spectrometry (MALDI-TOF MS) with a MALDI-7090 MS instrument (Shimadzu, Japan).

### Cell culture

Normal human bronchial epithelium cells BEAS-2B were maintained RPMI 1640 Medium (Hyclone, Life Technology, USA) supplemented with 10% fetal bovine serum (Hyclone, Life Technology, USA), and 1% penicillin/streptomycin at 37 °C in a 5% CO_2_ incubator. Murine bone marrow-derived dendritic cells (BMDC) were induced from bone marrow of BALB/c mice, the method was according to Lobo et al. [22]. In brief, bone marrow was cultured in RPMI 1640 medium containing 10% fetal bovine serum and 2 ng/mL of recombinant granulocyte–macrophage colony-stimulating factor (GM-CSF, PeproTech, USA). Media was changed every 3 days and supplemented with GM-CSF. Non-adherent cells were used and after 10 days.

### Cytotoxicity of the novel intranasal NE delivery system

The cytotoxicity of the NE, P22 and NE-P22 was tested in two cell lines using a Cell Counting Kit-8 (CCK-8, Dojindo, Japan). Briefly, BEAS-2B or BMDC cells in 100 μL RPMI 1640 medium were plated at a density of 10^4^ cells/well in flat-bottom 96-well plates and cultured in a humidified atmosphere containing 5% CO_2_ at 37 °C for 24 h. Then, 10 μL of NE, NE-P22 or free P22 were added to the wells at concentrations ranging from 3.125 to 50 μg/mL, or 10 μL of PBS was added as a control, and the cells were then incubated for another 24 h. Subsequently, 10 μL of CCK-8 was added to each well. Absorption at 450 nm was measured using an iMark microplate reader (Bio-Rad, USA). Cell viability was calculated according to the equation:

$${\text{Cell viability }}\left( \% \right)\, = \,\left( {{{{\text{OD}}_{\text{treatment}} } \mathord{\left/ {\vphantom {{{\text{OD}}_{\text{treatment}} } {{\text{OD}}_{\text{control}} }}} \right. \kern-0pt} {{\text{OD}}_{\text{control}} }}} \right)\, \times \, 100\% .$$

### NE-P22 uptake in lung epithelial cells

5 × 10^5^ BEAS-2B cells were seeded overnight on chambered coverglasses in 12-well plate. FITC-labeled P22 or NE-P22 were then added to cells to a final concentration of 20 μg/mL (for P22) for 1.5 h in media at 37 °C. Cells were washed with PBS, fixed for 20 min in 4% paraformaldehyde, and stained with DAPI (Life Technologies, USA). Cells were imaged on a LSM 780 confocal laser scanning microscope (CLSM, Zeiss, Germany).

### Induction of mice BMDC maturation

Mice BMDC were plated at 5 × 10^5^ per well in 6-well plates overnight before stimulation with antigen. Then cells were stimulated with P22, P22 + CpG, NE-P22 or NE-P22 + CpG. The concentration of P22 or CpG for each group are both 10 μg/mL. Cell cultures were incubated for 24 h and then harvested for flow cytometry analysis. Cells were collected, washed with PBS, then stained for 30 min at 4 °C with follow antibodies: APC-conjugated antibody anti-mouse CD11c, PE-conjugated antibody anti-mouse CD40, PE/Cy7-conjugated antibody anti-mouse CD80, APC/Cy7-conjugated antibody anti-mouse CD86 (BioLegend, San Diego, USA). Stained cells were washed with PBS, and then analyzed using a FACSVerse flow cytometer (BD, USA).

### In vitro release

The release profile of the NE-P22 and P22 were examined in PBS (pH = 6.8) at 37 °C as previously described [23]. In brief, 1 mL of sample (1000 μg/mL) was added to a preprocessed dialysis bag (molecular weight cutoff of 10,000 g/mol; Sangon, Shanghai, China) in 5 mL of PBS. Fifty-microliter samples were collected at 0, 0.25, 0.5, 1, 4, and 8 h. The concentration of P22 was detected by high performance liquid chromatography (HPLC) with a UV detector at a wavelength of 220 nm.

### In vivo release

Under anesthesia, BALB/c mice were intranasally administered 20 μL of the NE containing 20 μg of FITC-labeled P22 or 20 μg free FITC-labeled P22. After 0, 0.5, 1, 4 and 8 h, the mice were scanned using an IVIS system (Caliper Life Sciences, MA, USA). A background scan was also taken immediately before the intranasal administration to provide a threshold for adjusting the images collected at later timepoints.

### Antigen uptake in the nasal septa

The antigen uptake in the nasal septa was observed using epifluorescence microscope as previously described [24]. Briefly, mice were intranasally immunized with 10 μL per nare of 10 μg of FITC-labeled P22 alone or NE-P22 and sacrificed 2 h postinoculation. The nasal epithelium was isolated, frozen and sectioned using a CM 3050 S versatile cryostat (Leica, Germany). Slides were examined with an Eclipse 80i UV epifluorescence microscope (Nikon, Japan). Antigen uptake of APCs was analyzed using the method described by Tada et al. [25]. Briefly, P22-FITC uptake was analyzed using single cell suspensions isolated from nasal tissues of mice 6 h after intranasal immunized with NE-P22-FITC or free P22-FITC as previously mentioned. Cells were washed with staining buffer (BioLegend) and stained with PerCP/Cy5.5-anti mouse CD11c (BioLegend) or respective isotype control. The samples were analyzed using a FACSVerse flow cytometer (BD, USA).

### Protective effect of the novel intranasal NE delivery system

#### Immunization

For intranasal immunization, mice were immunized four times at 1-week intervals with 20 μL (10 μL per nostril) of the NE, NE-P22, NE-P22 with CpG, P22 with CpG 1826 (Invitrogen, Shanghai, China), P22 only, NE with CpG or CpG only. The dosage of P22 or CpG is 20 μg per mice respectively.

#### *H. pylori* infection and determination of colonization

One week after the final immunization, the mice were challenged orally four times with 2 × 10^8^ CFU of BALB/c mouse-adapted *H. pylori* at 1-day intervals as we previously performed [26]. Four weeks after the last infection, the mice were euthanized, the stomachs were cut along the greater curvature. Half of the stomach was collected, fixed with 4% paraformaldehyde for 24 h and then embedded in paraffin. Each specimen of all of the tested mice was sectioned for three slides and stained with hematoxylin and eosin. The inflammation of all slides was evaluated and graded independently by two pathologists according to the criteria. The inflammation scores depend on the infiltrate of inflammatory cells, epithelial hyperplasia, and mucous cell metaplasia, among other factors, similar to the criteria in previous study [27]. The remaining half of stomach was used to determine IgA levels using enzyme linked immunosorbent assay (ELISA) and colonization of *H. pylori* in the stomach using real-time quantitative PCR. An analysis of *H. pylori* 16S rDNA was performed according to previously described methods [28, 29]. The spleens of mice were collected for enzyme-linked immunospot assay (ELISPOT) and cytokines assays, and the serum of mice was gathered to determine IgG, IgG1 and IgG2a levels using ELISA.

#### ELISPOT

ELISPOT assays were conducted using a mouse IFN-γ precoated ELISPOT kit (Dakewe, Beijing, China). In brief, 5 × 10^5^ lymphocytes isolated from immunized mouse spleens were incubated with 10 μM peptide in RP-10 at 37 °C overnight. The plates were then washed and incubated with biotinylated anti-IFN-γ monoclonal antibodies at 37 °C for 1 h. The plates were washed again and incubated with streptavidin–horseradish peroxidase for 1 h at 37 °C. Finally, the plates were washed, and the spots were revealed using AEC buffer. The spots were counted, and the results are presented as the number of spot-forming cells/5 × 10^5^ spleen cells.

#### Measurement of cytokines secretion by splenocytes

Measurement of cytokines secretion by splenocytes were performed according to previous studies [30, 31]. 5 × 10^6^ lymphocytes isolated from immunized mouse spleens were seeded in 24-well tissue culture plates in 500 μL of DMEM/F12 (Hyclone, Life Technology, USA) with l-glutamine supplemented with 10% fetal calf-serum and 50 mM 2-mercaptoethanol (Sigma, USA). Following 72-h stimulation with P22 (10 μg/mL), supernatants were collected following centrifugation and analyzed by ELISA for IL-4, IL-17A, IL-22 and IFN-γ concentration (Dakewe. China).

#### Statistical analysis

Experimental data are expressed as the mean ± standard deviation (mean ± SD) deviation. Differences between treatment groups were analyzed using unpaired Student’s t-tests. Other data were analyzed using one-way ANOVA followed by Newman–Keuls tests for dependent variables. All data were analyzed using GraphPad Prism 5.01 software. p < 0.05 was considered statistically significant.

## Results

### Preparation of the novel intranasal NE delivery system

#### Influence of different Smix ratios on size, zeta potential and PDI

**Fig. 1:**
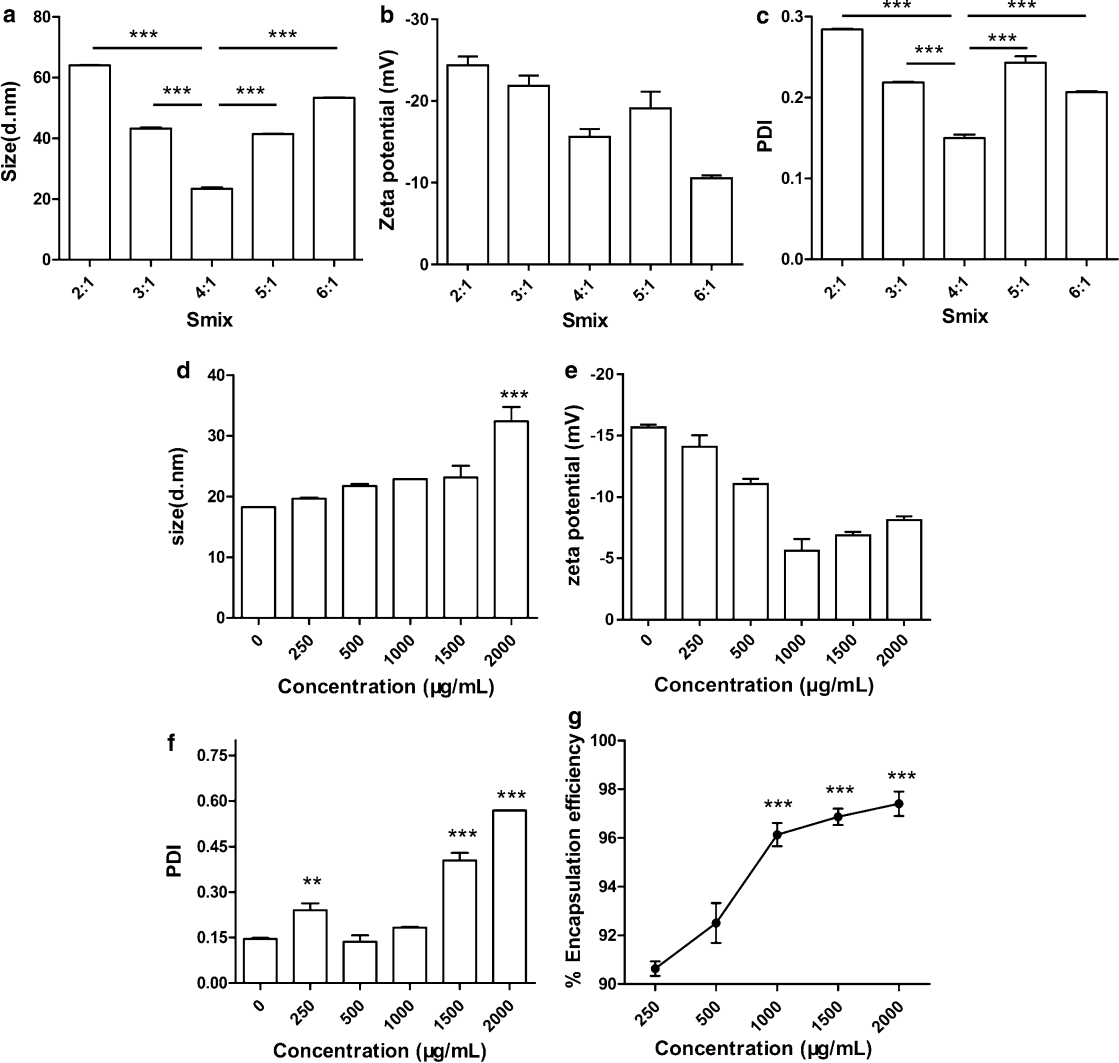
Influence of different Smix ratios and the P22 concentration on characterizations. **a**–**c** Influence of different Smix ratios on size, zeta potential and PDI of nanoemulsions: **a** particle sizes. **b** Zeta potentials. **c** Polydispersity indexes. **d**–**g** Influence of the P22 concentration on size, zeta potential, PDI and encapsulation efficacy: **d** particle sizes. **e** Zeta potentials. **f** Polydispersity indexes. **g** Encapsulation efficiencies. For **d**–**f** the results obtained were compared with blank nanoemulsion (epitope peptide concentration = 0). For **g**, the results obtained were compared with 250 μg/mL group. The data are expressed as the mean ± SD (n = 3). *p < 0.05, **p < 0.01, ***p < 0.001

To evaluate the influence of the Smix ratio (Surfactant: Co-surfactant, w/w), five nanoemulsions with different Smix ratios (2:1, 3:1, 4:1, 5:1 and 6:1) were prepared. NEs ranged in size from 23.4 to 64.0 nm (Fig. 1a), and they were spherical with zeta potentials ranging from − 10.6 to − 24.4 mV (Fig. 1b) and polymer dispersity index (PDI) values ranging from 0.150 to 0.284 (Fig. 1c). Among these formulations, the 4:1 Smix ratio showed the lowest PDI (0.150 ± 0.007) and the smallest particle size (23.4 ± 0.8 nm), indicating that this NE had a close size distribution, and its zeta potential was − 15.6 ± 1.69 mV. Based on these data, the 4:1 Smix ratio was the best formulation for this novel NE system.

#### Influence of the P22 concentration on size, zeta potential, PDI and encapsulation efficacy

The effects of the P22 concentration on the particle size PDI, and zeta potential are shown in Fig. 1d–f. The particle size increased slightly as the P22 concentration rose from 250 to 1500 μg/mL, ranging from 18.23 to 23.12 nm, but it jumped to 32.39 ± 4.09 nm at a P22 concentration of 2000 μg/mL (Fig. 1d). The zeta potential changed from − 14.10 to − 5.61 mV as the P22 concentration increased from 500 to 1000 μg/mL, but it was stable within the range of − 5.61 to − 8.12 mV as the P22 concentration increased from 1000 to 2000 μg/mL (Fig. 1e). The PDI positively correlated with the P22 concentration (from 500 to 2000 μg/mL), except at 250 μg/mL, and we observed that the PDI increased rapidly when the P22 concentration was increased from 1000 to 1500 μg/mL (Fig. 1f). Importantly, we found that as the P22 concentration was increased from 250 to 1000 μg/mL, the encapsulation efficiency increased from 90.62 to 96.13% then stabilized at approximately 96% (96.13% to 97.40%) in the P22 concentration range of 1000 to 2000 μg/mL (Fig. 1g). Considering the dispersibility, particle size and encapsulation efficiency, we chose a peptide epitope concentration of 1000 μg/mL to use with the optimal Smix ratio (4:1).

### Basic characteristics and stability of the NE system

**Fig. 2:**
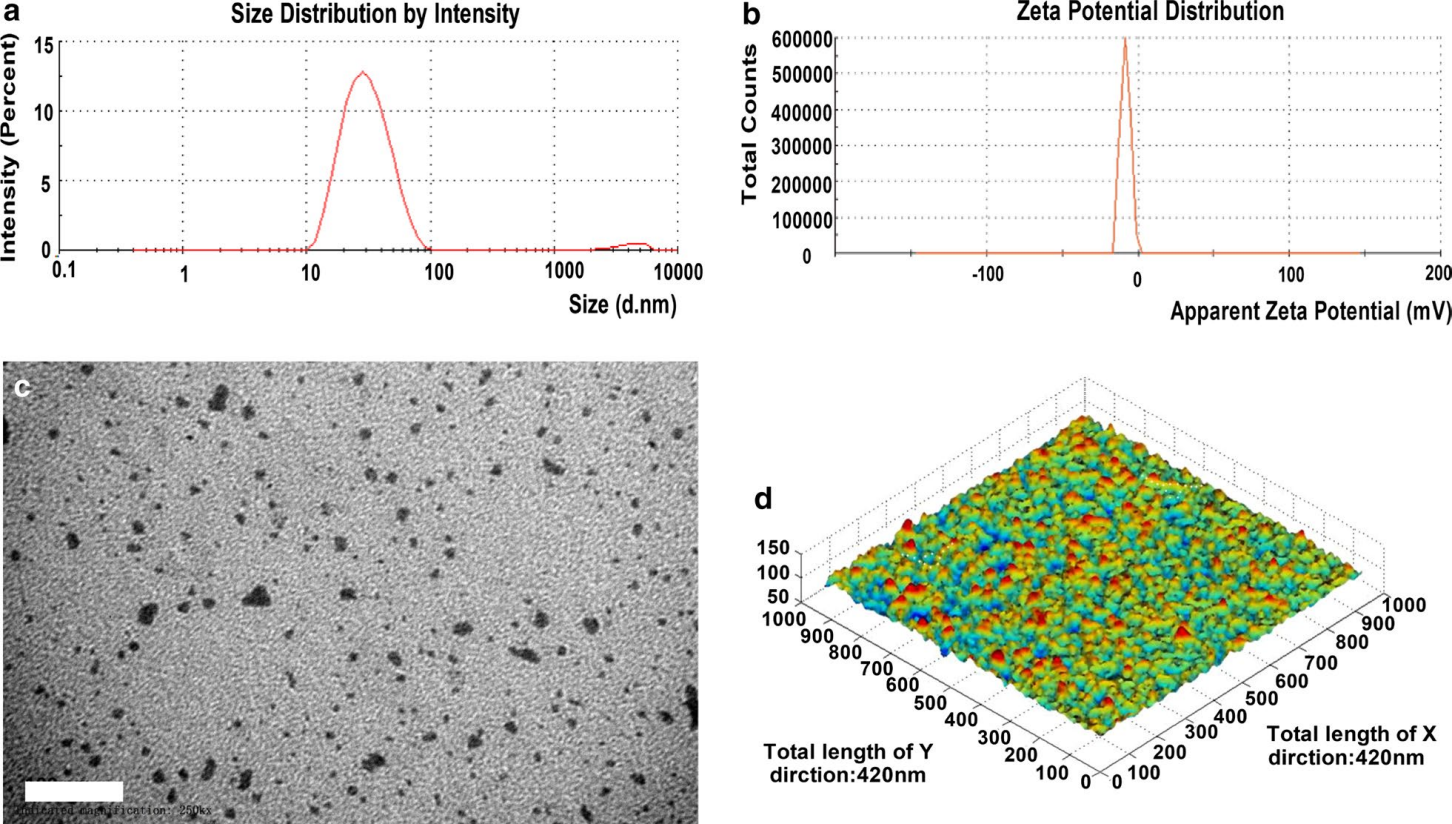
Physical characteristics of the NE-P22 vaccine. **a** Size diameter and distribution. **b** Zeta potential and distribution. **c** Transmission electron micrograph (TEM), scale bar = 100 nm. **d** Atomic force microscopy (AFM) micrograph, the X and Y axes both have a total length of 420 nm

The preparation of the NE system was complete when the liquid became clear, at which point it had a mean particle size of 22.84 ± 0.01 nm with a zeta potential of − 5.168 ± 1.687 mV (Fig. 2a, b). TEM images showed that the droplet sizes were within the range of approximately 1–100 nm, which indicated that the system had a relatively uniform distribution of particle sizes, as shown in Fig. 2c. The molecular morphology of this novel NE was further examined using AFM (Fig. 2d), which revealed that the droplets of this novel system exhibited a spherical morphology with a mean diameter of approximately 20 nm, and no droplet aggregation was observed. Consistently, these results suggested that the particles of this novel system have a relatively uniform size distribution and a spherical morphology. To evaluate the stability of P22 in this novel system, a blank NE and the P22-containing system were examined by MALDI-TOF MS (Additional file 1: Fig. S1a–c). The highest main peak of 1435 m/z was observed for both the epitope peptide and the novel NE system, indicating that P22 was stable during preparation.

### Cytotoxicity of the NE delivery system

Cytotoxicity is one of the most important factors that could limit the use of the NE delivery system. In this study, the cytotoxic effects of the novel system were tested in BEAS-2B and BMDC cells via CCK8 assays after 24 h of incubation. The NE demonstrated no toxicity against BEAS-2B and BMDC cells after 24 h. The cell survival ratios for the NE system, which contained 0.9% NaCl and was diluted 20-fold, were 96.25 ± 9.01% and 94.46 ± 11.86% (grade 1, low toxicity) in the BEAS-2B and BMDC, respectively (Additional file 1: Fig. S2), with dilutions increasing from 2-fold (the maximum dilution producing no effect on cell growth, 500 μg/mL) to 8-fold (31.25 μg/mL) based on the standard ISO 10993-5:1999 Part 5 Test for in vitro cytotoxicity. When the concentration of P22 was 50 μg/mL, i.e., when the blank NE and NE-P22 were each diluted 20 times, neither of the nanoemulsions had an impact on the viability of these cell lines. This finding suggests that both P22 and this delivery system have relatively low cytotoxicity.

### Cellular Uptake of the NE delivery system by normal human bronchial epithelium cells

**Fig. 3:**
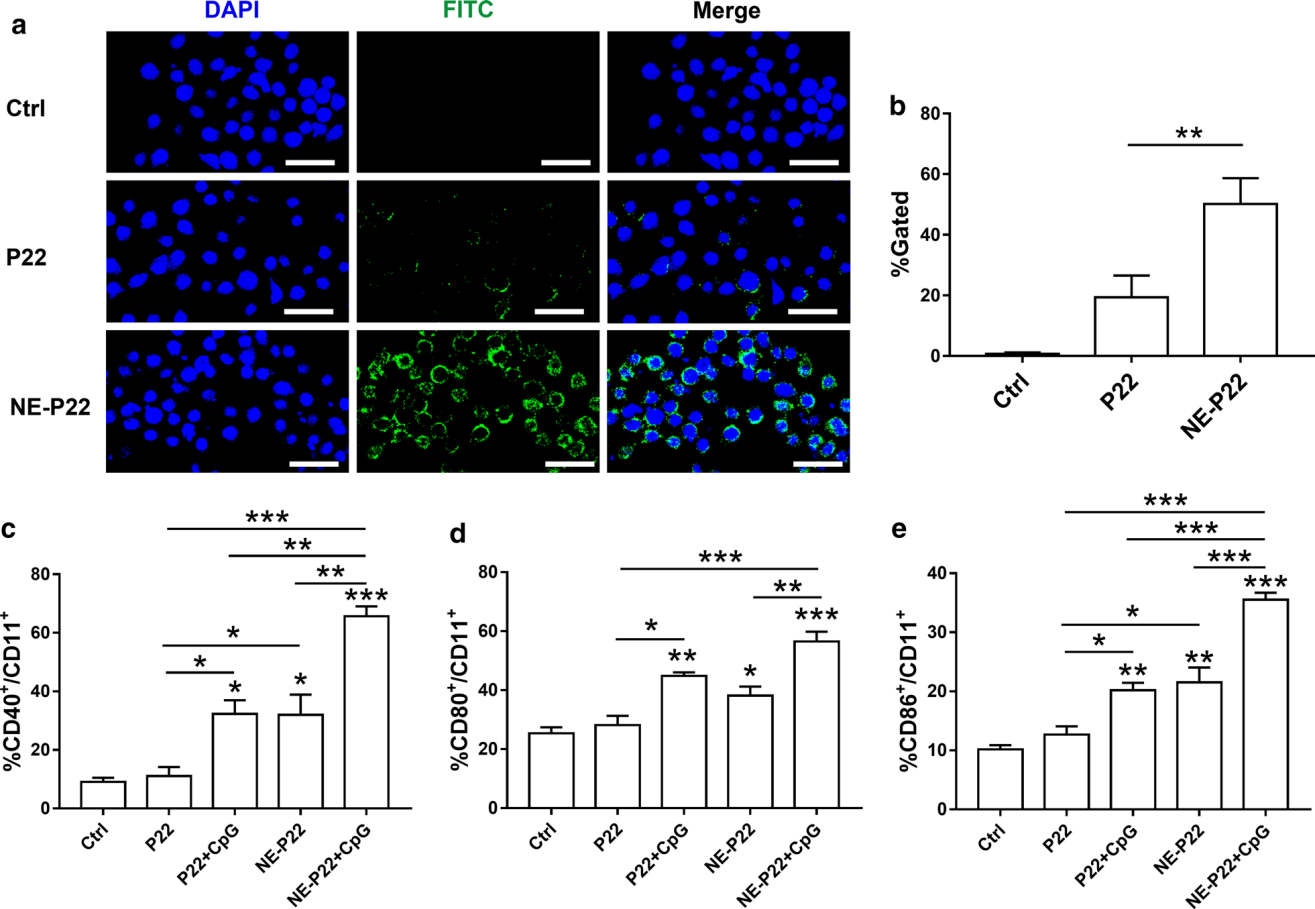
In vitro cellular uptake of NE-P22 and induction of bone marrow dendritic cells maturation. **a** In vitro confocal fluorescence imaging of BEAS-2B cells treated 1 h with P22 or NE-P22, PBS was used as control, P22 was labeled with FITC (green fluorescence) and nuclei were stained with DAPI (blue fluorescence) (Scale bar, 50 μm). **b** Uptake of FITC-labeled P22 by BEAS-2B cells were analyzed using flow cytometry (n = 3). **c**–**e** Relative proportions of mice bone marrow dendritic cells positive for (**c**) CD40, **d** CD80 and **e** CD86 surface markers were measured using flow cytometry. The data are expressed as the mean ± SD (n = 3). *p < 0.05, **p < 0.01, ***p < 0.001

Cellular uptake efficiency of the NE delivery system was evaluated by using BEAS-2B cells, a normal human bronchial epithelium cell line. As shown in Fig. 3a, CLSM images revealed more FITC-labeled P22 was captured by BEAS-2B cells in NE-P22 group than P22 group. This result was further corroborated via flow cytometry (Fig. 3b), the rates of FITC+ cells are 19.38 ± 12.49% and 50.17 ± 14.76% for P22 and NE-P22 group, separately. These results suggest that the NE delivery system effectively enhanced the epitope peptides uptake efficiency.

### Induction of bone marrow dendritic cells maturation

We next measured the ability of the NE system to induce the maturation of dendritic cells by detecting co-stimulatory molecules of mice BMDC, such as CD40, CD80 and CD86, via flow cytometry. The rates of CD40^+^/CD11^+^ (Fig. 3c), CD80^+^/CD11^+^ (Fig. 3d) and CD86^+^/CD11^+^ (Fig. 3e) cells are significantly increased in P22 + CpG and NE-P22 group, moreover, NE-P22 + CpG induced the maturation of BMDC to a much greater extent than the same concentration of P22 + CpG or NE-P22. Flow cytometry data of cell surface markers of BMDC are in Additional file 1: Fig. S3. These results suggest that the NE delivery system can promote the maturation of BMDC and can play a synergistic role with CpG.

### Sustained release of the NE system in vitro and in vivo

**Fig. 4:**
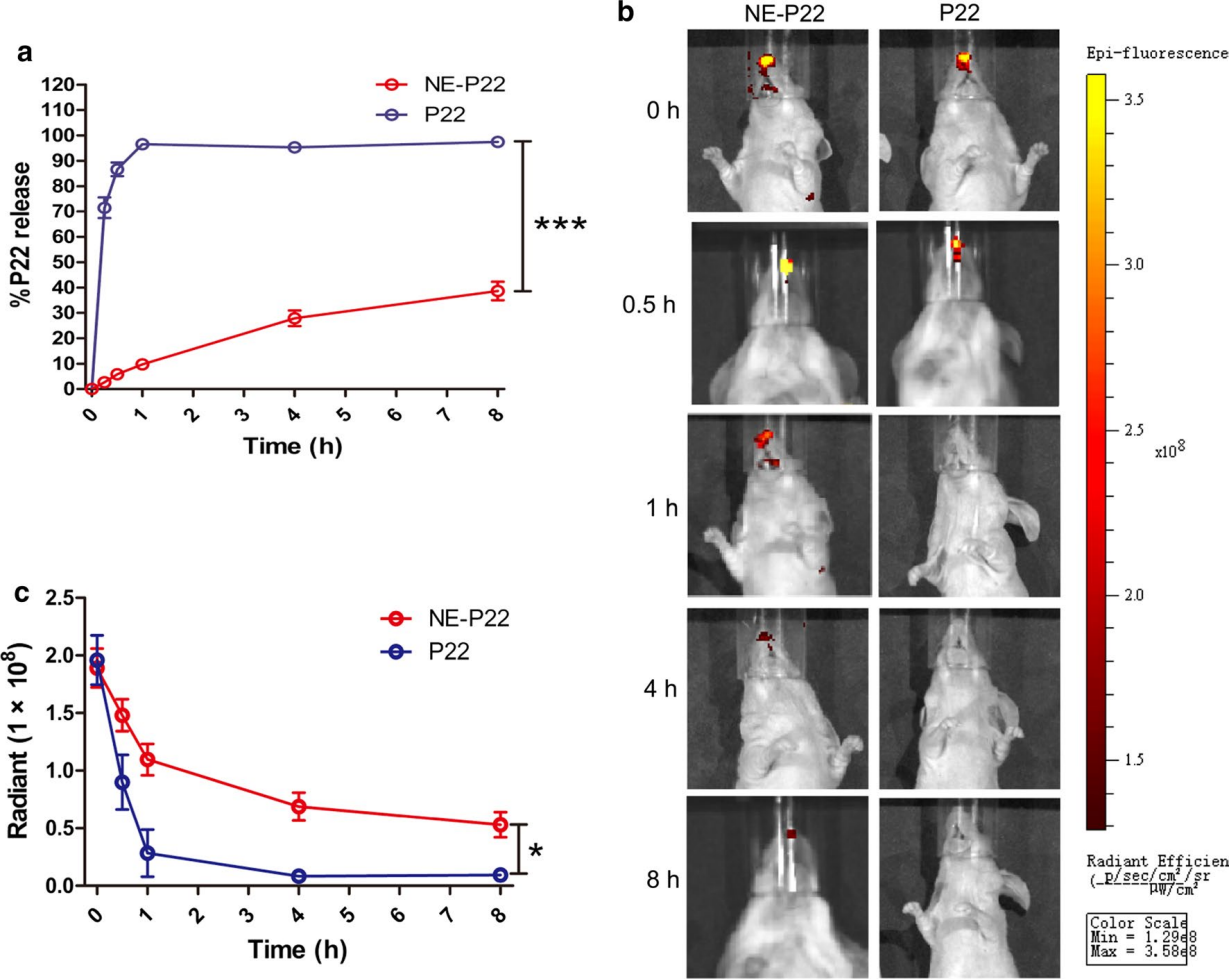
In vitro and in vivo release of NE-P22. **a** In vitro release profile of NE-P22. **b** In vivo fluorescence imaging of FITC-labeled P22 in the mouse nasal cavity, relative fluorescence intensity recorded at 0, 0.5, 1, 4, and 8 h after nasal administration of NE-P22 or P22. **c** Quantitation of fluorescence intensity. The data are expressed as the mean ± SD (n = 3). *P < 0.05, ***P < 0.001

The in vitro release profile of P22 and the NE system was assessed. We clearly observed that the release of P22 alone is exponentially faster than that of the NE system. The cumulative release of P22 and the NE system in PBS was 86.60 ± 4.67% and 5.84 ± 1.86%, respectively, after 30 min (Fig. 4a). In addition, the release percent of P22 was 3.5-fold and 2-fold that of the NE-P22 in PBS after 4 h and 8 h, respectively. In conclusion, this novel system displays sustained-release characteristics, which is significantly different from the in vitro release behavior of P22 (p = 0.001, p < 0.01).

To evaluate the nasal residence time of a water solution and the NE system, FITC-labeled P22 was prepared, and an IVIS was used to detect the content of FITC-labeled P22 in vivo. P22 and the NE system were intranasally administered to mice, which were monitored after 0, 0.5, 1, 4 and 8 h. Representative results are presented in Fig. 4b. Fluorescence decreased in the nasal cavity for both free P22 and the NE system over time, but the decrease occurred more slowly for the NE (Fig. 4c) such that its quantitative fluorescence, even 8 h after administration, was higher than that of the water solution after 1 h. These results suggest that this system can slow the release of P22 in vitro, significantly prolong nasal residence time in vivo.

### Antigen uptake in the nasal septa

**Fig. 5:**
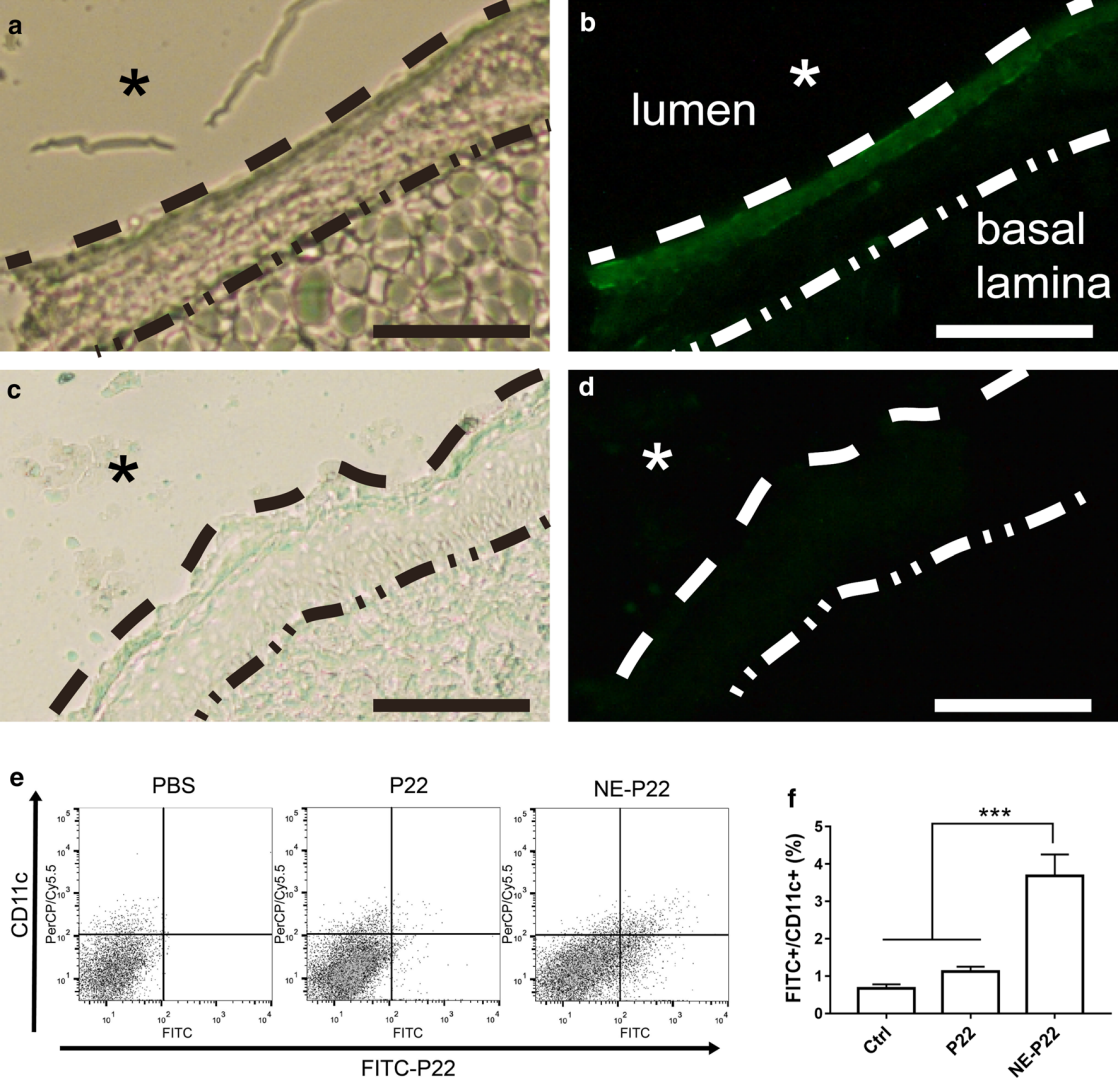
Antigen uptake in the nasal septa. Micrograph of frozen sectioned nasal septal tissue isolated from mice immunized with **a**, **b** NE-P22 or **c**, **d** P22 alone, P22 was labeled with FITC. Features indicated: septal columnar epithelium (between dashed lines), basal lamina (dash-dotted line), epithelial-luminal junction (dashed line), and nasal lumen asterisk. **a** and **c** are light micrographs, **b** and **d** are corresponding fluorescence micrographs (Scale bar, 50 μm). **e**, **f** BALB/c female mice were immunized intranasally with PBS, P22-FITC, or NE-P22-FITC. The single cell suspensions were prepared from nasal tissues on 6 h post immunization. The isolated cells were stained with PerCP/Cy5.5-anti-mouse CD11c mAb or respective isotype control. P22-FITC uptake was analyzed by flow cytometry. The data are expressed as the mean ± SD (n = 3). ***P < 0.001

We next evaluated the effect of NE delivery system on the antigen uptake in the nasal epithelium in vivo. Mice were intranasally immunized with FITC-labeled free P22 or NE-P22 and sacrificed 2 h post inoculation. Nasal septal tissue was isolated and the frozen sections were observed via epifluorescence microscope. As shown in Fig. 5a–d, immunization with free P22 resulted in diffuse background fluorescence (Fig. 5c, d). In contrast, immunization with NE-P22 resulted in more intense fluorescence on the mucosa epithelium area (Fig. 5a, b), reflecting antigen uptake into the nasal epithelium, which indicated that the NE delivery system can facilitate epitope peptides uptake across the mucosal layer in vivo. Nevertheless, in this study, we have found that NE-P22 effectively enhanced P22 uptake by CD11^+^ DCs in nasal mucosal tissues (Fig. 5e, f), indicating that the NE system would be useful as an epitope peptide delivery vehicle to APCs.

### The NE delivery system promotes cellular immune responses

To investigate the immune responses by which our system enhances the protective efficiency of the epitope vaccine against *H. pylori* infection in vivo, we evaluated the P22-induced antigen-specific immune responses including antibody repertoire and cytokine release profiles.

**Fig. 6:**
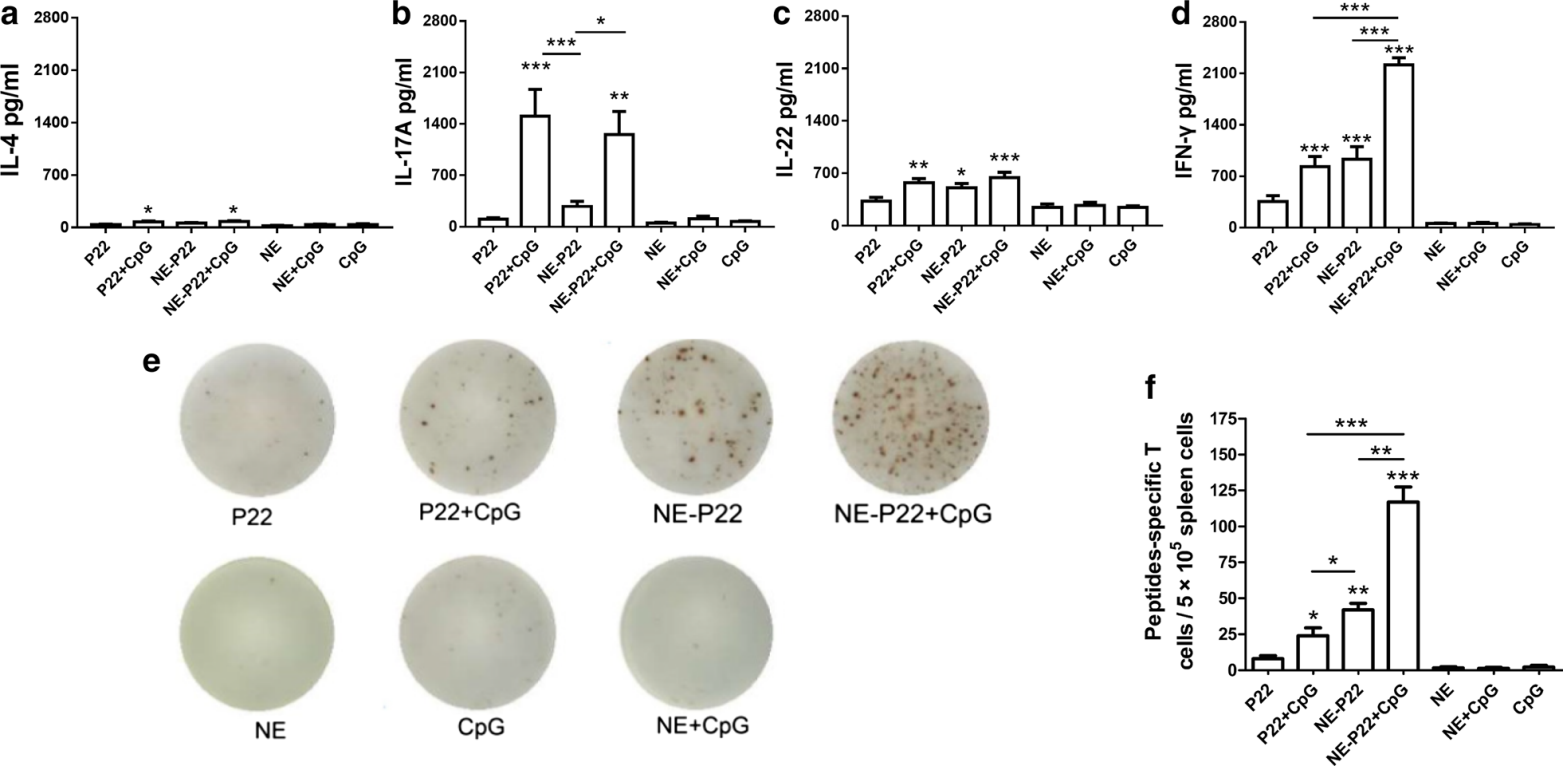
NE-P22 stimulated stronger specific Th1 responses. Comparison of the release profile of **a** IL-4, **b** IL-17A, **c** IL-22 and **d** IFN-γ in the supernatant of the ex vivo stimulated splenocytes of mice intranasally immunized with indicated preparations. **e** Representative ELISPOT results of the indicated preparations. **f** The number of P22 specific IFN-γ-producing cells in the spleen after immunized with indicated preparations. The results obtained were compared with CpG. The data are expressed as the mean ± SD (n = 5). *p < 0.05, **p < 0.01, ***p < 0.001

However, there were no differences in serum IgG, IgG1, IgG2a or gastric IgA levels between the groups (Additional file 1: Fig. S4a–d). We next evaluated the cytokine release profiles of immunized mice splenocytes after antigen stimulated. Figure 6a–d showed four cytokine profiles of Th2-bias cytokine (IL-4, Fig. 6a), Th17-related cytokine (IL-17A, Fig. 6b), Th22-related cytokine (IL-22, Fig. 6c) and Th1-skewed cytokine (IFN-γ, Fig. 6d). IL-4 slightly increased in P22 + CpG and NE-P22 + CpG group compared with the CpG control group (1.87- and 1.94-fold, respectively). IL-17 increased in P22 + CpG and NE-P22 + CpG group (21.3- and 17.8-fold, respectively). IL-22 mildly increased in P22 + CpG, NE-P22 and NE-P22 + CpG group (2.36-, 2.07- and 2.65-fold, respectively). IFN-γ, in particular, dramatically increased in P22 + CpG, NE-P22 and NE-P22 + CpG group (18.8-, 21.1- and 50.2-fold, respectively). In addition, the IFN-γ level of NE-P22 + CpG group is significantly higher than P22 + CpG and NE-P22 group (p < 0.001).

We further evaluated the frequency of peptide P22-specific IFN-γ-producing cells in spleens. As shown in Fig. 6e, f, intranasal immunization with free P22 peptide induced approximately 8 P22-specific IFN-γ-producing cells per half million spleen cells, which is not significantly different from that of the CpG control group. However, the frequency of P22-specific IFN-γ-producing cells was significantly increased in P22 + CpG, NE-P22 and NE-P22 + CpG group. Among these, NE-P22 stimulated a relatively stronger Th1 response than did P22 + CpG, but NE-P22 + CpG induced the most robust Th1 response in mice.

### Protective effect of the NE system

To estimate the effect of our NE delivery system on the protective efficiency of the epitope vaccine against *H. pylori* infection in vivo, we intranasally immunized mice with NE-P22, NE-P22 with CpG (NE-P22 + CpG), P22 with CpG (P22 + CpG) or P22 only. Mice immunized with CpG only, blank NE or NE plus CpG (NE + CpG) were used as controls. The mice were then challenged with *H. pylori* and euthanized at the indicated times to evaluate the bacterial load of *H. pylori* in their stomachs and the pathological inflammation of gastric tissue.

**Fig. 7:**
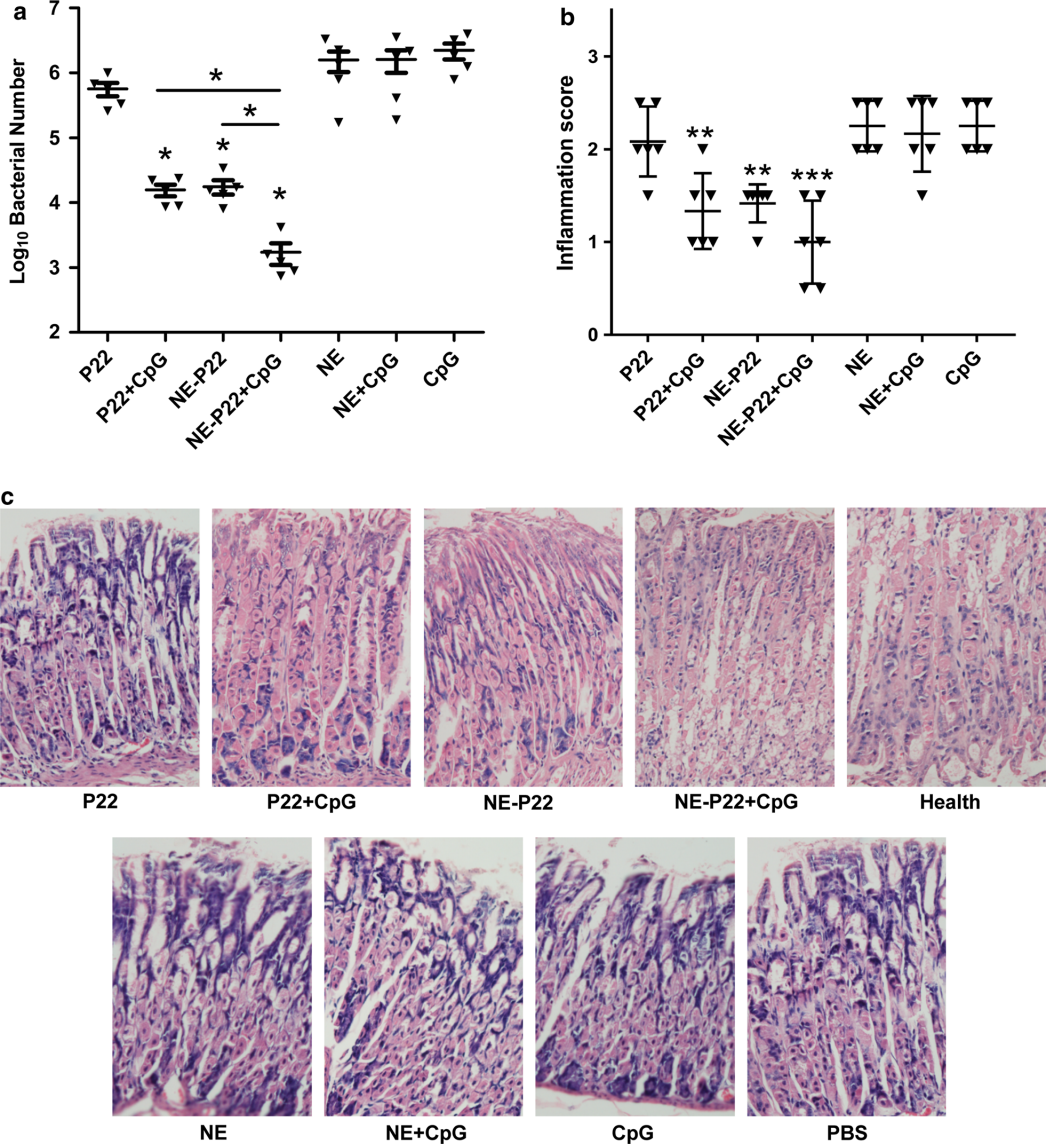
Intranasal immunization with NE-P22 protected mice against *H. pylori* infection. **A** Colonization of *H. pylori* in mice intranasally immunized with P22, P22 plus CpG, NE-P22, NE-P22 plus CpG, NE, NE plus CpG, CpG. The results obtained were compared with CpG. The colonization of *H. pylori* was quantified by real-time PCR 4 weeks post-challenge (n = 5). **b** Gastric inflammation scores of mice immunized with peptides were identified 4 weeks after infection (n = 6) and **c** representative gastric histopathology of immunized, PBS controls and health mice are shown (H&E staining, original magnification ×200). The data are expressed as the mean ± SD. *p < 0.05, **p < 0.01, ***p < 0.001

As shown in Fig. 7a, mice immunized with NE-P22 or P22 + CpG had significantly lower bacterial loads compared with those of the CpG group, while mice immunized with NE-P22 + CpG had the lowest bacterial loads. For pathological inflammation of gastric tissue, there were only mild inflammatory neutrophil and mononuclear-cell infiltrates of P22 + CpG, NE-P22 and NE-P22 + CpG immunized mice, whereas CpG controls showed moderate foci of neutrophil and mononuclear-cell infiltration (Fig. 7b, c). Moreover, mice immunized with NE-P22 + CpG showed the slightest inflammation in their stomach. These results suggest that our NE delivery system enhances the protective efficiency of the epitope vaccine against *H. pylori* infection, especially in the presence of CpG.

## Discussion

This study is attempt to enhance efficiency of intranasal epitope vaccine against *H. pylori* based on an optimized nanoemulsion delivery system. We previously identified several naturally occurring immunodominant Th1 cell responses to HpaA in *H. pylori*-infected subjects and then systematically characterized the immunodominance hierarchy of HpaA-specific Th1 cell responses in mice during vaccination [26, 32], and this specific Th1 cell response can protect mice from *H. pylori* infection but not other type of bacteria (such as *Pseudomonas aeruginosa* and *Staphylococcus aureus*, Additional file 1: Fig. S5). The HpaA154-171 (P22)-specific Th1 cell response was demonstrated to be the most immunodominant response [7]. Studies of viral, bacterial, and tumor immunity have demonstrated that immunodominant T cells are highly effective in host adaptive immunity against pathogens [33, 34]. The focusing of immune responses against highly conserved epitopes may be crucial for the prevention and treatment of infections [35]. However, low immunogenicity is a major drawback of epitope peptides [36]. Thus, approaches to increase the immunogenicity of epitope peptides are needed.

Vaccine delivery systems that mimic pathogenic particles to increase the immunogenicity of antigens are widely used [37]. Among these delivery systems, emulsions have proven to be an effective and safe strategy. As an emulsion, MF59 has been approved as an adjuvant for influenza vaccines by the Food and Drug Administration (FDA) for several years [38]; however, it still has some drawbacks, such as poor adjuvant effects due to the thermodynamic instability of its emulsion system as well as an average particle size that exceeds 160 nm. In addition, MF59 immunogens generate an insufficiently active cellular immune response [39]. NEs have been confirmed to be efficiently taken up by the mucosal lymphoid tissue to deliver encapsulated antigens to antigen-presenting cells (APCs) after nasal immunization [40]. Subsequently, they can boost immunity and generate specific immune responses [41]. In a previous study, our NE adjuvant system can enhance the mucosal immunity response elicited by intranasal immunization with a protein antigen [20]. The data of the present study also show that this NE is an ideal delivery system. Therefore, in this study, we firstly attempted to design a novel intranasal NE delivery system to enhance the immunogenicity of the P22 epitope peptide.

Herein, we designed and prepared an optimized nasal NE system with a mean particle size of 22.84 ± 0.01 nm when loaded with 1000 μg/mL epitope peptide P22, and the particles exhibited a spherical morphology. Current research has shown that particles smaller than 300 nm are the most effective at crossing the mucosal layer [42], while particles of smaller sizes, such as 20–45 nm, are more likely to migrate efficiently to lymph nodes than larger ones [43]. External energy is required for the formation of NE systems, and two main methods for the preparation of such nanoemulsions exist, dispersion (high-energy methods) and condensation (low-energy methods). The dispersion or high-energy methods involve an energy input provided by high-shear stirring, high-pressure homogenizers, or ultrasound generators. However, to obtain small droplet-sized NEs, a substantial amount of mechanical energy is needed, making this preparation route unfavorable for industrial application. The condensation or low-energy methods make use of the phase transitions that occur during the emulsification process due to changes in the spontaneous curvature of the surfactant [44]. These low-energy methods are particularly attractive due to their mild preparation conditions, but they are not yet well understood with respect to the required surfactant composition and the structural pathway of formation [45]. Therefore, the novel NE system developed in this study was prepared using a one-step low-energy emulsification method, and it was designed to have a relatively optimal size based on the above previous understandings.

In contrast to soluble peptide antigens, droplets of the proper size are inefficiently taken up and presented by APCs, whereas pathogen-sized particles and protein aggregates may be efficiently taken up by APCs [43]. Our NE system has been tested as an effective delivery system to increase the antigen uptake activity of BEAS-2B normal human bronchial epithelium cells in this study (Fig. 3a, b), these results indicated that this NE system could exhibit beneficial effects of internalization and enhance epitope-specific immune responses. Furthermore, we investigated the ability of this NE system to facilitate maturation of dendritic cells, due to the DCs maturation process is important for the immune response. Mature dendritic cells express high levels of co-stimulatory molecules such as CD40, CD80 and CD86. NE-P22 + CpG induced the maturation of BMDCs to a greater extend than P22, P22 + CpG or NE-P22 (Fig. 3c–e). Recent research found that the surface charge of nanomaterials can effect on the efficacy of the antigen uptake. Some research suggested that polymer-based micro- and particularizes with positive charge will facilitates the antigen uptake. However, there are emulsion adjuvant such as MF59 [46, 47], nanoemulsion with negative charge also promotes recruitment of immune cells, including antigen presenting cells, which are facilitated to engulf antigen and transport it to draining lymph node where the antigen is accumulated [48]. At the same time, our previously study found that nanoemulsion vaccine with negative charge may easily be captured by the APC cells [49]. In this study, the result which nasal NE system with negative charge significantly facilitated the uptake of epitope in the nasal septa is similar in these research focus on the negative charge. In short, this system could effectively increase the antigen uptake ability and facilitate maturation of APCs.

Antigen load and kinetics are essential for evoking efficient protective immune responses [50]. For example, during HPV infections, too little antigen exposure results in a failure to elicit a protective T cell response [51]. There are two strategies widely used to prolong the exposure of the immune system to an antigen; the first is to increase the frequency of vaccination, while the other is to prolong the residence time of the antigen in the vaccination site or in germinal centers [50]. In this study, the NE system clearly exhibited a sustained-release ability in vitro (Fig. 4a), and it significantly prolonged the nasal residence time of the epitope peptide P22 and facilitated the epitope uptake in nasal septa and CD11^+^ DCs in nasal tissues (Figs. 4b, c, 5a–f). However, whether this system can prolong the residence time of the epitope peptide in germinal centers should be further investigated. Furthermore, prolonged antigen exposure improves long-lived protective T-cell memory in mice [52]. Therefore, it is assumed that enough antigen exposure is the key for the induction and maintenance of protective memory [51]. In our subsequent work, we will focus on the ability of this system to promote the maintenance of protective memory.

Some materials, such as Toll-like receptor (TLR) ligands, have been added into vaccines as components of adjuvants. These adjuvant components can help to polarize immune responses [53]. Among the TLR9 ligands, CpG ODNs are widely used in vaccinology to enhance T-cell responses to recombinant antigens [54], and Th1 cells have been confirmed to play a vital role in the elimination of *H. pylori* [55–57]. We also previously demonstrated that immunization with immunodominant epitopes plus CpG can recruit specific Th1 lymphocytes into the gastric mucosa to promote the elimination of *H. pylori* colonization [26, 58]. In this study, our data showed that NE-P22 + CpG can elicit dramatically more potent protective immune responses than P22 + CpG (Figs. 6a–f, 7a–c).

Thus, we hypothesize that there are synergistic interactions between this system and CpG. The signaling cascade triggered by the interaction of TLR9 with CpG proceeds by stimulating MyD88, IRAK and TRAF-6. Subsequently, the recruitment of various MAP kinases and transcription factors (including NF-κB, AP1 and IRF-7) upregulates the expression of proinflammatory genes [59]. These Th1 cytokines (such as IFN-γ) then contribute to the improvement of the CD8+ T-cell response by inducing the maturation of plasmacytoid dendritic cells [60]. A previous study showed that the intranasal administration of an antigen with W805EC, a NE adjuvant, induces a MyD88-independent antibody response and a MyD88-dependent Th-1 and Th-17 cell-mediated immune response [61]. In this study, we found that intranasally immune with NE-P22 + CpG can dramatically increase the Th17-related cytokine IL-17A and Th1-skewed cytokine IFN-γ, but Th2-bias cytokine IL-4 or Th22-related cytokine IL-22, secretion levels of splenocytes after P22 stimulation (Fig. 6a–d), the results are similar to those of previous studies [61]. However, in this study, the Th17-related cytokine IL-17A levels of P22 + CpG and NE-P22 + CpG are comparable, consistent with the results of bacterial loads and inflammation levels (Fig. 7), we consider that induction of Th1 immune response is the key factor of this NE system to enhance the protection effect against *H. pylori* infection.

In summary, we designed a NE system that offers high vaccine efficacy via nasal administration without obvious cytotoxicity. This NE system significantly prolonged the nasal residence time of the epitope peptide, and the NE-P22 vaccine without CpG induced potent specific Th1 responses that reduced bacterial colonization. Furthermore, the protection efficacy was significantly enhanced when the NE-P22 vaccine was combined with CpG. These findings demonstrate the promise of this novel NE system for the intranasal delivery of an *H. pylori* epitope vaccine. However, only one epitope peptide was used in this NE vaccine, whereas a proper vaccine may require several epitopes to elicit a well-rounded protective immune response. Thus, we need to identify additional immunodominant epitope peptides to expand the *H. pylori* vaccine arsenal. In addition, given the interspecies differences between mice and humans, the immunodominant epitope of mice may not match that of humans. Differences may even exist between individuals [62], in which case vaccines with personalized epitope peptides may be an optimized strategy.

## Conclusion

We developed a novel NE system with an optimal Smix ratio of 4:1 and an epitope peptide concentration of 1000 μg/mL. The optimal NE vaccine had a mean particle size of 22.84 ± 0.01 nm and exhibited high vaccine efficacy after nasal administration without obvious cytotoxicity. As expected, the NE system significantly prolonged the nasal residence time of the epitope peptide to over 8 h and facilitated the uptake of epitope in the nasal septa. Immunization with the NE-based P22 vaccine induced potent specific Th1 responses and reduced bacterial colonization without CpG. Furthermore, the protective efficacy of the NE-P22 vaccine was further enhanced when it was administered with a CpG adjuvant. These findings demonstrate the promise of this novel NE system for the intranasal delivery of an *H. pylori* epitope vaccine.

## Additional file

**Additional file 1.** Supplementary document.
