# Supplementary material for: Epitope-loaded nanoemulsion delivery system with ability of extending antigen release elicits potent Th1 response for intranasal vaccine against Helicobacter pylori
Source: J Nanobiotechnology. 2019 Jan 19;17:6. doi: 10.1186/s12951-019-0441-y (PMC6339695; doi:10.1186/s12951-019-0441-y)
Supplement: Supplementary file 1 — Additional file 1. Supplementary document. [file 12951_2019_441_MOESM1_ESM.docx]

**Epitope-loaded nanoemulsion delivery system with ability of extending antigen release elicits potent Th1 response for intranasal vaccine against *Helicobacter pylori***

Yun Yang^1^, Li Chen^1,2^, Hong-wu Sun^1*^, Hong Guo^3^, Zhen Song^1^, Ying You^1^, Liu-yang Yang^1^, Ya-nan Tong^1^, Ji-ning Gao^4^, Hao Zeng^1^, Wu-chen Yang^1, 3, 5*^, Quan-ming Zou^1*^.

^1^National Engineering Research Center of Immunological Products, Department of Microbiology and Biochemical Pharmacy, College of Pharmacy, Third Military Medical University, Chongqing, China; ^2^Department of Blood Transfusion, The Second Affiliated Hospital, Third Military Medical University, Chongqing, China; ^3^Department of Gastroenterology, The Second Affiliated Hospital, Third Military Medical University, Chongqing, China; ^4^ Institute of Combined Injury of PLA, College of Military Preventive Medicine, Third Military Medical University of Chinese PLA, Chongqing. ^5^Department of Hematology, The Second Affiliated Hospital, Third Military Medical University, Chongqing, China.

*Corresponding authors.

E-mail of authors:

Yang Yun (yy9008@hotmail.com), Li Chen (chenli200401@163.com), Hong-wu Sun (sunhongwu2001@163.com), Hong Guo (hguoxqyy@163.com), Zhen Song (743500658@qq.com), Ying You (280256057@qq.com), Liu-yang Yang (yang3608@foxmail.com), Ya-nan Tong (470455006@qq.com), Ji-ning Gao (jngao@163.com), Hao Zeng (zeng1109@163.com), Wu-chen Yang (seasonwcy@126.com), Quan-ming Zou (qmzou2007@163.com).


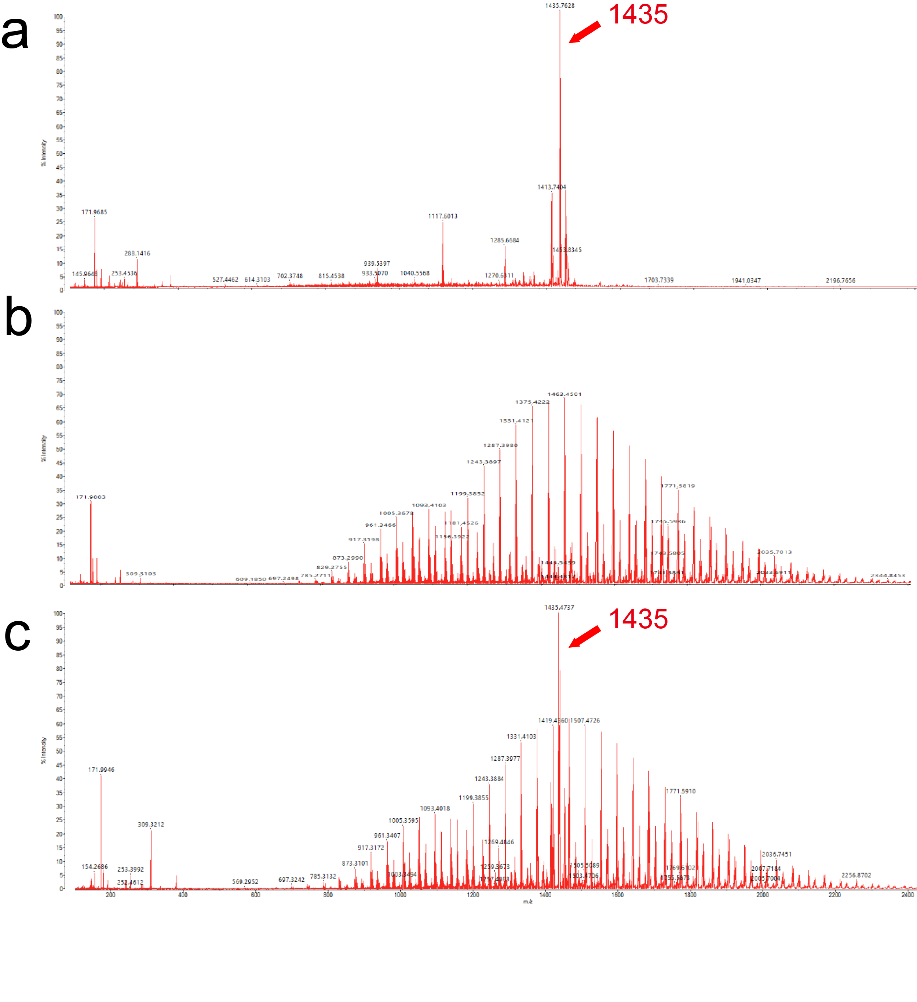


**Fig S1. Stability of epitope peptide in the NE system.**

**Note:** (**a**) Free P22, (**b**) blank NE and (**c**) NE-P22 were analyzed *via* MALDI-TOF mass spectrometry. The main peak of P22 (m/z=1435) was indicated by the red arrow.


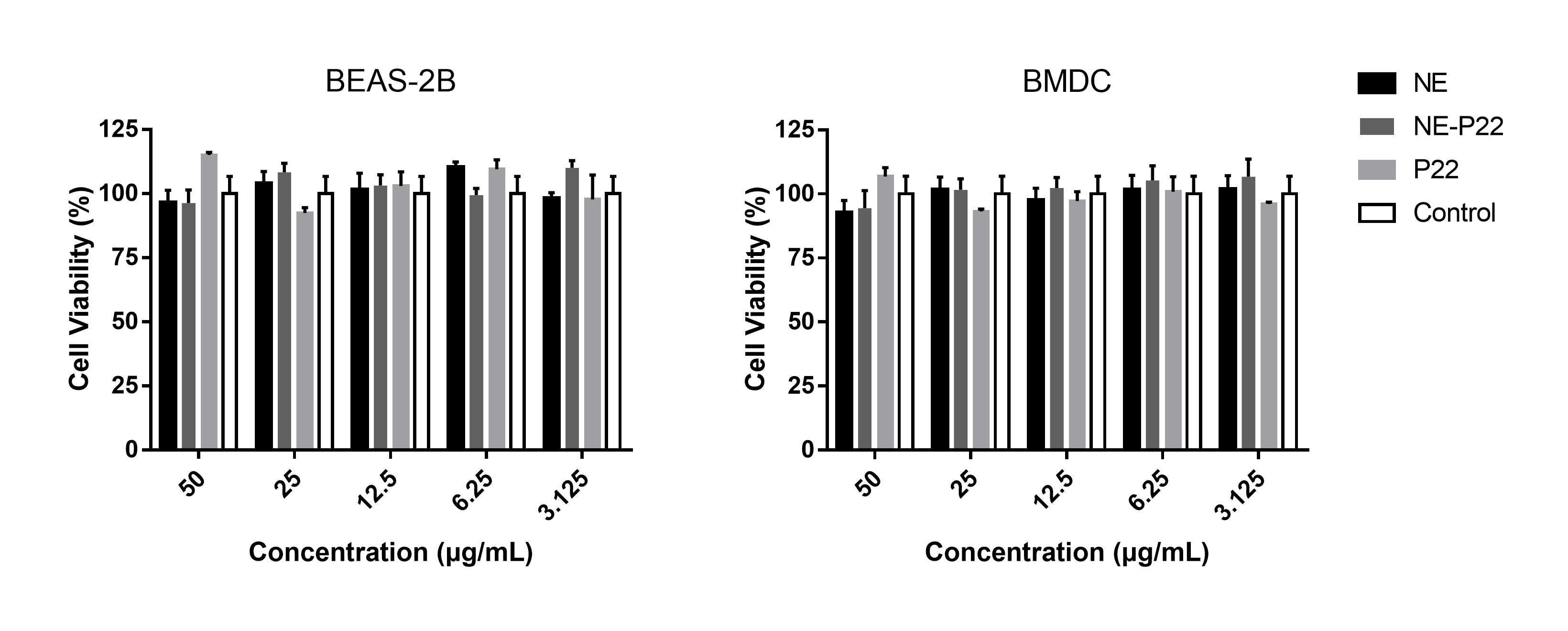


**Fig S2. *In vitro* cytotoxicity of the NE system.**

**Note:** Relative viability of BEAS-2B and BMDC cells in culture exposed to different peptide concentrations of P22, NE-P22 and blank NE respectively for 24 h, blank NE was diluted to the same dilution of NE-P22. PBS was used as a control. The data are expressed as the mean ± S.D (n = 3).


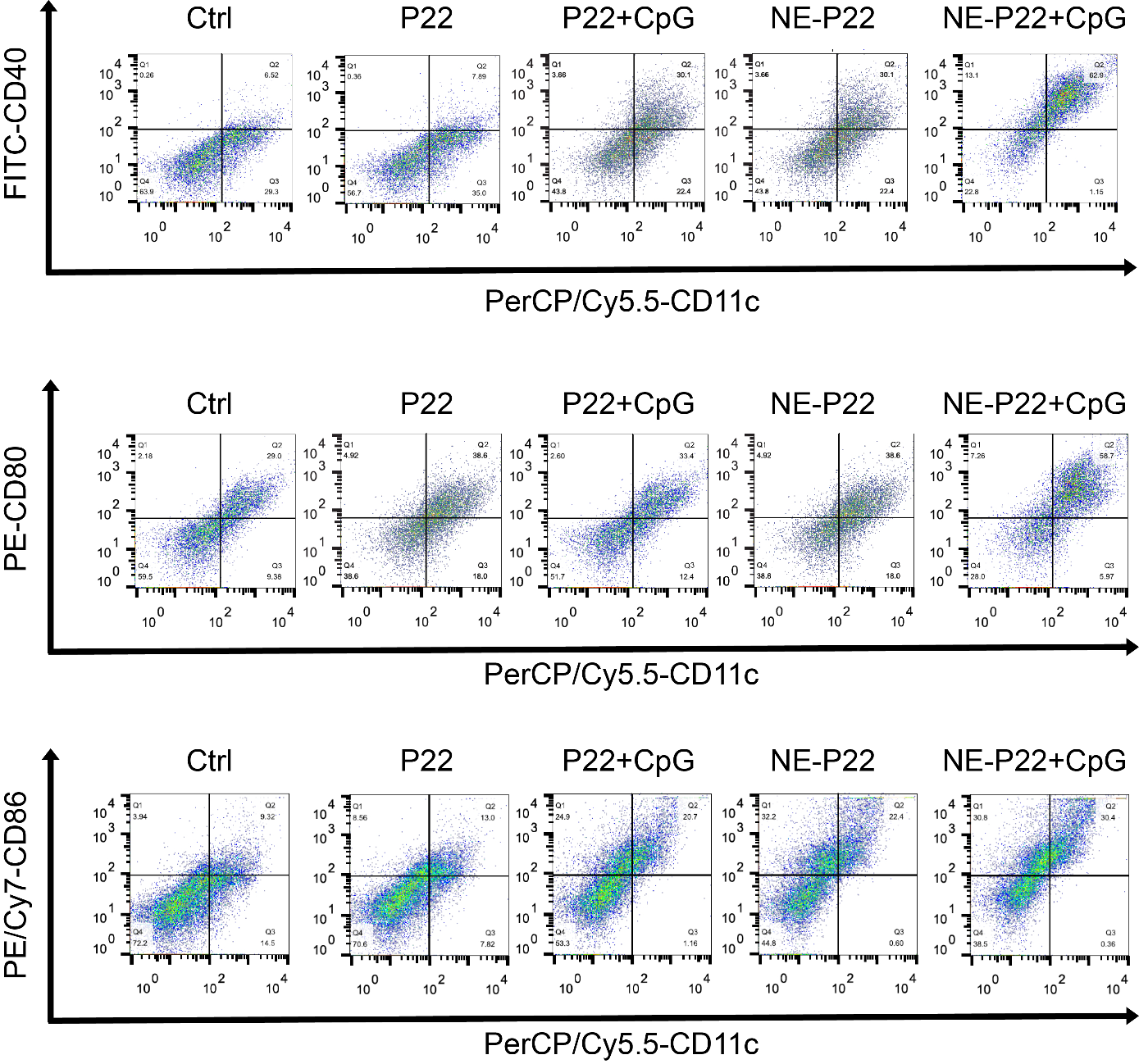


**Fig S3. Flow cytometry data of cell surface markers of BMDC cells.**


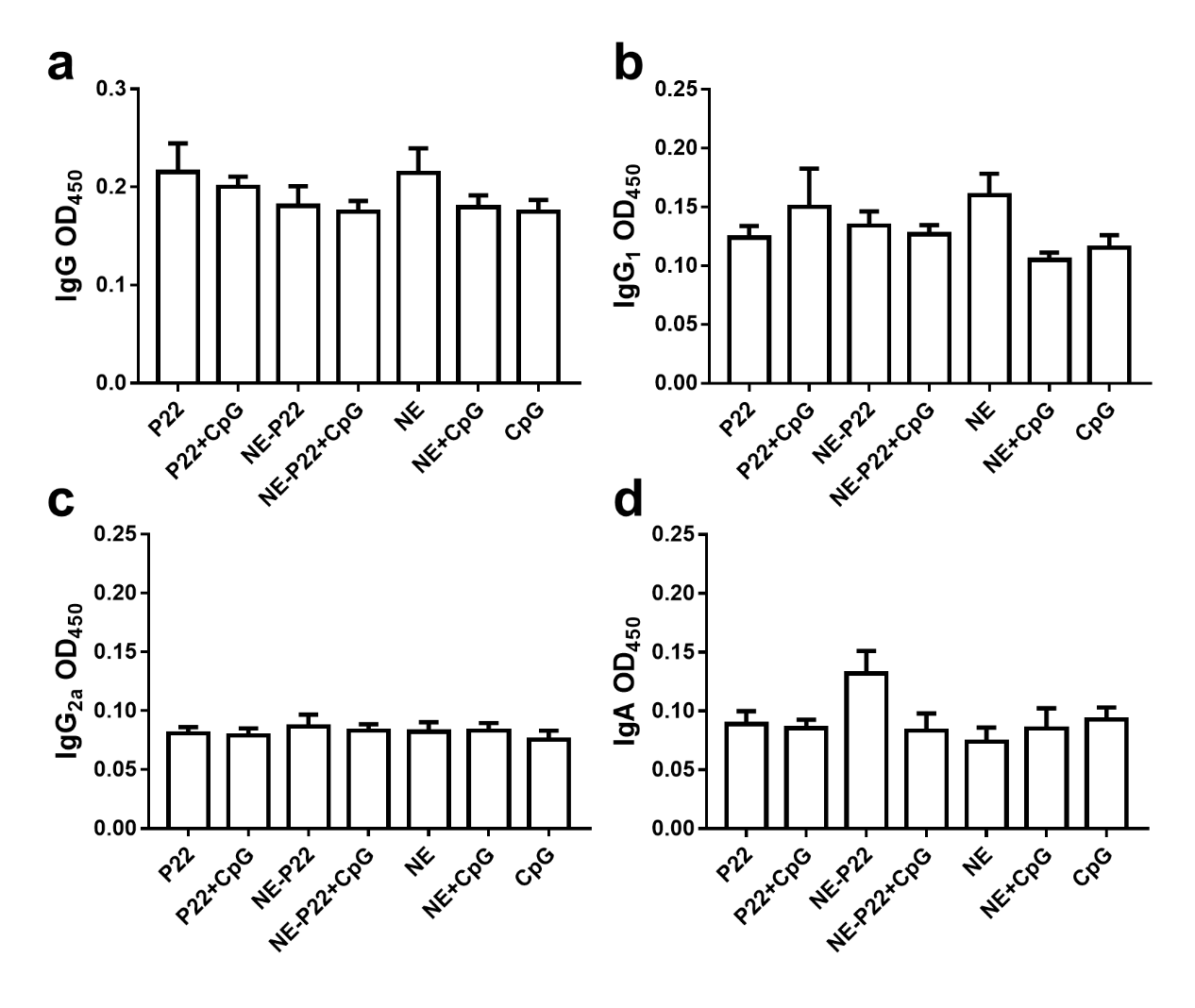


**Fig S4. Humoral and mucosal immune antibody levels**

**Note:** Humoral and mucosal immune antibody levels after nasal immunization of mice with the indicated preparations. Serum levels of (**a**) IgG, (**b**) IgG1 and (**c**) IgG2a were assayed after 50-fold diluted. (**d**) Levels of IgA in gastric were assayed. The results obtained were compared with CpG.


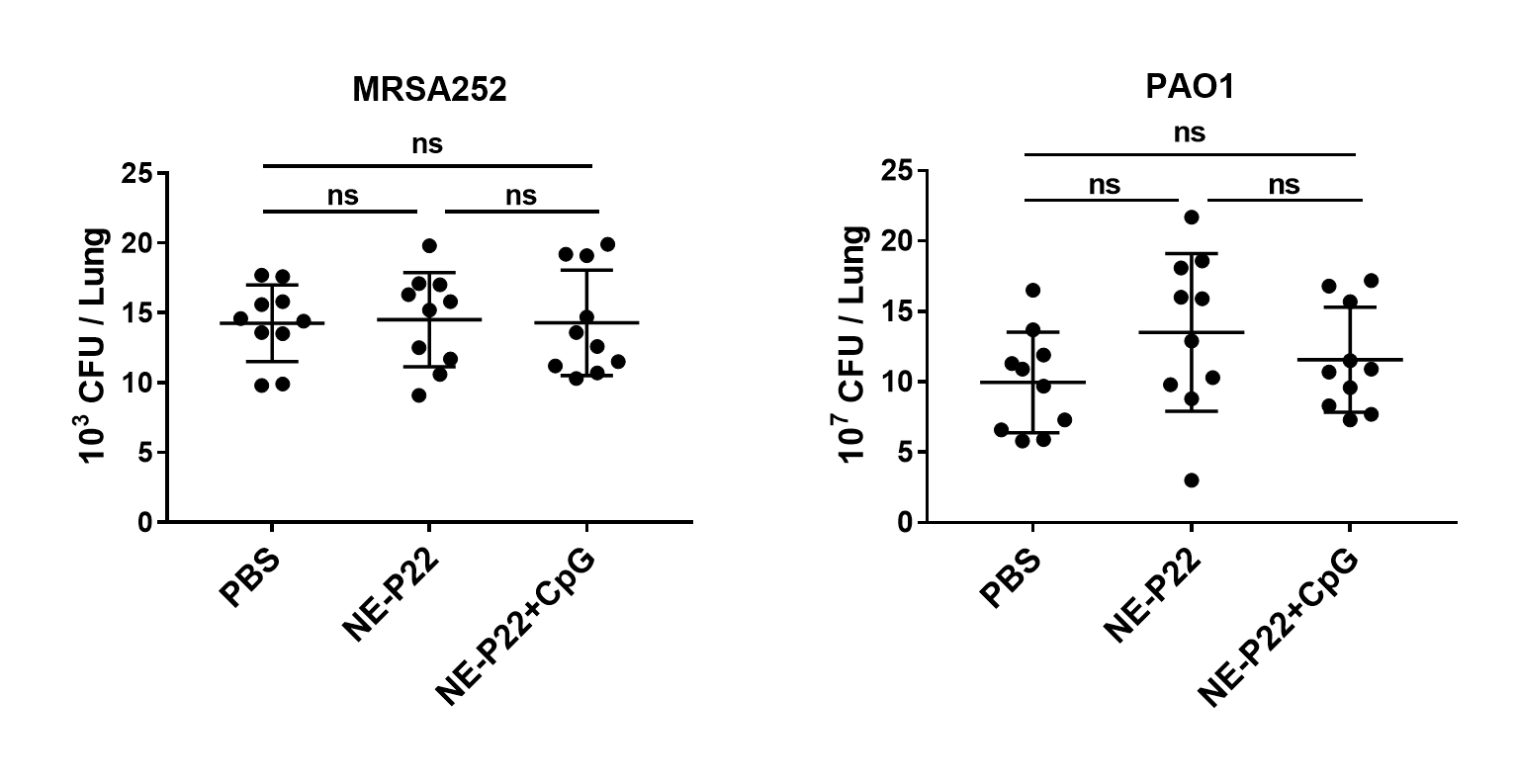


**Fig S5. Humoral and mucosal immune antibody levels**

**Note:** Mice were immunized with PBS, NE-P22 or NE-P22+CpG as we mentioned in the manuscript. One week after the final immunization, the mice were intranasally inoculated with 4×10^8^ CFU MRSA 252 (*Staphylococcus aureus*) or 5×10^6^ CFU PAO1 (*Pseudomonas aeruginosa*). Mice were sacrificed 24 hours after infection, their lungs were collected for bacteria burden analysis. The data are expressed as the mean ± S.D. (n = 10). ns, no significant difference.
